# Infectious diseases burden of health system collapse in Gaza and Lebanon: a call for action

**DOI:** 10.1016/j.lanepe.2026.101811

**Published:** 2026-08-07

**Authors:** Giuseppe Pipitone, Guido Granata, Souha S. Kanj

**Affiliations:** a Infectious Diseases Unit, ARNAS Civico-Di Cristina Hospital, Palermo, Italy; b Infectious Disease Service, Fondazione Policlinico Universitario Campus Bio-Medico, 00127, Rome, Italy; c Division of Infectious Diseases, Department of Internal Medicine, and Center for Infectious Diseases research (CIDR), Faculty of Medicine, American University of Beirut, Lebanon

Armed conflicts destroy health infrastructure, pathogen surveillance, and create conditions, such as overcrowding, displacement, contaminated water, malnutrition, absence of diagnostics and infection control, and supply chain failure, in which pathogens prone to causing outbreaks thrive.^1^ Gaza and Lebanon represent two currently documented such severe collapses. We summarize the epidemiological evidence and identify priority actions for the global infectious diseases community.

In Gaza, the collapse of water, sanitation, and hygiene (WASH) infrastructure is the primary driver of infectious diseases risk. As of 2024, 89% of WASH infrastructure has been destroyed, and only 17% of water supply remains operational (often with water and sewage mixing).^2^ As of August 2025 81% of WASH facilities were within Israeli-militarized zones or under displacement orders, leaving more than one million people with less than 6 L of drinking water daily, and 500,000 people without enough water for hygiene, and 44% of population had acute watery diarrhea.^2^^,^^3^ Scabies, pediculosis, and skin infections have become highly endemic, and the risk of vaccine-preventable diseases is higher.^2^ Acute malnutrition is extimated to affect more than 100,000 children and 37,000 pregnant women,^4^ causing an increase in immune alteration and increased susceptibility to infectious diseases.

Of 36 hospitals, only 18 remain partially functional, and more than 1700 healthcare workers have been killed since October 2023, the highest toll recorded from a single conflict.^2^^,^^3^ As consequence, an independent population-representative household survey estimated 75,200 violent deaths through January 2025 alone, above the contemporaneous Ministry of Health estimation, together with 8540 excess non-violent deaths above pre-conflict projections.^5^ More than 18,500 patients, including 4096 children, currently await medical evacuation for conditions that cannot be managed locally, while the humanitarian corridor to the West Bank and East Jerusalem remains largely closed.^3^

In addition, chronic traumatic stress in childhood and the elderly impairs innate and adaptive immunity, we could hypothesize that these alterations could significantly increase the susceptibility to micro-organisms. Furthermore, bombardment has generated an estimated 39 million tons of debris containing silica, asbestos, polyvinyl chloride, and combustion particulates, to which two million people, half of them children, have been exposed for more than two years during critical windows of pediatric lung development.^6^ The persistent airway hyperreactivity could lead to not only chronic pulmonary diseases, but also the so-called “siege pollution exposure respiratory syndrome” (SPERS), for which no pulmonary health infrastructure currently exists in Gaza. The result would cause everlasting debilities.^6^

In Lebanon, the renewed escalation compounds a health system already weakened by the 2019 economic collapse, the Beirut Port blast and the COVID-19 pandemic. Within ten days of the onset of the Israeli military operations in Lebanon, 47 primary healthcare centers and five hospitals were forced to suspend activities, adding displacement to a population already hosting over 1.5 million Syrian refugees since 2011, and thousands of Palestinian refugees.^7^ Hospital occupancy had already reached 70% before the crisis; essential-medicine stockouts affected up to 50% of facilities; and in many areas, primary care had been reduced to two operational days per week.^7^ As of June 14, 2026, 172 attacks on emergency medical services had killed 133 health-care workers and injured 402, with 79 attacks on health care affecting 20/153 (13%) hospitals leading to closures or partial operations, finally 63 primary care centers were affected and 51 forced to close.^8^ Approximately 1.2 million people are displaced, many in crowded collective shelters which favor the spread of infections such as scabies, measles, influenza, hepatitis A, cholera, and multi-drug-resistant tuberculosis. A recent cholera outbreak^9^ and a report of transmission of NDM-5-carrying *Escherichia coli* between refugees, host communities and the environment are a reminder of how weak infrastructures can lead to spread of infectious diseases.^10^ In hospitals receiving war-wounded patients, wound infections with multidrug-resistant Gram-negative pathogens, particularly *Acinetobacter baumannii*, are frequently untreatable and act as a source of nosocomial spread to patients with comorbidities and immunodeficiency.

We call on Global Health researchers, clinicians, and professional societies to act on three priorities. First, to help healthcare workers in Gaza and Lebanon to document outbreaks as real-time reports. Second, we ask for the restoration of humanitarian health access—supply of vaccines, good quality antimicrobials, oral rehydration solutions, and therapeutic nutrition—as a non-negotiable prerequisite for epidemic prevention and control. Third, to establish longitudinal cohort studies to characterize the immunological consequences of early-life exposure to conflicts and wars and to prospectively document the SPERS burden in Gaza and Lebanon's children over the next decade.

Finally, because none of these measures is achievable amid active wars, we join other health societies in calling for an immediate cessation of hostilities as the essential pre-condition for restoring disease surveillance, immunization, and water and sanitation systems; for the unimpeded entry of medical supplies, vaccines, nutritional support, and health personnel; and for the protection of healthcare facilities and staff, whose destruction is in itself a principal driver of the infectious diseases burden described here.

## Contributors

GP: conceptualization, data curation, writing original draft, writing-review and editing, validation, visualization; GG: conceptualization, writing original draft, writing-review and editing, supervision, validation, visualization; SSK: data curation, writing original draft, writing-review and editing, supervision, validation, visualization.

## Declaration of interests

GG declares no conflict of interest. SSK declares receiving speaker honoraria from Pfizer, Menarini, MSD, and Antabio, and serving as President of the International Society of Antimicrobial Chemotherapy and former President of the Lebanese Society of Infectious Diseases and Clinical Microbiology. GP declares sponsorship to attend congress from Pfizer and Tillotts, and serving as Board Member of the Italian Society of Infectious and Tropical Diseases and Treasurer of the Executive Committee of the ESCMID Study Group for Infections in the Elderly (ESGIE).
